# Phylogenetic Analysis Reveals Common Antimicrobial Resistant *Campylobacter coli* Population in Antimicrobial-Free (ABF) and Commercial Swine Systems

**DOI:** 10.1371/journal.pone.0044662

**Published:** 2012-09-12

**Authors:** Macarena P. Quintana-Hayashi, Siddhartha Thakur

**Affiliations:** Department of Population Health and Pathobiology, College of Veterinary Medicine, North Carolina State University, Raleigh, North Carolina, United States of America; Unidad de Microbiología, Facultad de Medicina Universidad Rovira, Spain

## Abstract

The objective of this study was to compare the population biology of antimicrobial resistant (AR) *Campylobacter coli* isolated from swine reared in the conventional and antimicrobial-free (ABF) swine production systems at farm, slaughter and environment. A total of 200 *C. coli* isolates selected from fecal, environmental, and carcass samples of ABF (*n* = 100) and conventional (*n* = 100) swine production systems were typed by multilocus sequence typing (MLST). Sequence data from seven housekeeping genes was analyzed for the identification of allelic profiles, sequence types (STs) and clonal complex determination. Phylogenetic trees were generated to establish the relationships between the genotyped isolates. A total of 51 STs were detected including two novel alleles (*glnA* 424 and *glyA* 464) and 14 novel STs reported for the first time. The majority of the *C. coli* isolates belonged to ST-854 (ABF: 31, conventional: 17), and were grouped in clonal complex ST-828 (ABF: 68%, conventional: 66%). The mean genetic diversity (*H*) for the ABF (0.3963+/−0.0806) and conventional (0.4655+/−0.0714) systems were similar. The index of association (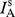) for the ABF (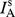 = 0.1513) and conventional (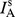 = 0.0991) *C. coli* populations were close to linkage equilibrium, indicative of a freely recombining population. Identical STs were detected between the pigs and their environment both at farm and slaughter. A minimum spanning tree revealed the close clustering of *C. coli* STs that originated from swine and carcass with those from the environment. In conclusion, our study reveals a genotypic diverse *C. coli* population that shares a common ancestry in the conventional and ABF swine production systems. This could potentially explain the high prevalence of antimicrobial resistant *C. coli* in the ABF system in the absence of antimicrobial selection pressure.

## Introduction

*Campylobacter* is one of the leading causes of foodborne diarrheal illness in the U.S. and a significant public health concern worldwide [1]. The majority of human cases are attributed to *C. jejuni*, although human infections due to *C. coli* are estimated to be underreported [2]. Food animals like poultry, cattle and swine are common hosts and important reservoirs of *Campylobacter* species. Swine are known to be a main animal reservoir of *C. coli* [3],while *C. jejuni* is prevalent in poultry [4] and cattle [5].

The use of antimicrobials in food animals for disease treatment, prophylaxis, and to enhance animal growth [6], [7] has been under scrutiny due to associations with the development of resistant bacteria to important antimicrobials in human medicine [8]. Antimicrobial resistant (AR) *Campylobacter* have been shown to be shed by swine, poultry, and cattle reared in conventional production systems that use antimicrobials for treatment and growth promotion [9]–[11]. However, studies have demonstrated that AR *Campylobacter* strains are also prevalent in farm animals from antimicrobial-free (ABF) and organic production systems [12], [13]. Concerns of *C. coli* as a zoonotic pathogen have been highlighted due to the presence of AR strains in higher frequency than *C. jejuni* [14]. *C. coli* from swine have exhibited a diversity of multidrug-resistant (MDR) profiles that persist throughout the production stages at farm and slaughter, even in the absence of antimicrobial selection pressure [11]. The prevalence of *C. coli* strains exhibiting higher frequency of resistance to antimicrobials than *C. jejuni* further highlights the importance of conducting molecular epidemiologic studies of this zoonotic pathogen.

Multilocus sequence typing (MLST) has been a valuable technique for the determination of epidemiological associations between human *Campylobacter* cases and sources of infection [15], as well as for the characterization of bacterial population structures [16]. MLST schemes have been developed for *C. jejuni* [16] and *C. coli* [17] among various bacterial species, indexing variation in seven housekeeping genes. This method has several advantages, among which are the generation of portable data, and the availability of a centralized internet database [18]. Previous work performed by our laboratory have used MLST for molecular epidemiologic studies of *C. coli* in swine [3], [19], [20], and for the genotyping of human and retail meat isolates [21].

We recently reported the high prevalence of MDR *C. coli* from pigs reared in conventional and ABF production systems at farm, slaughter, and the environment. To better understand the persistence of MDR *C. coli* strains in swine production, we proposed to genotypically characterize the *C. coli* population in the ABF and conventional swine production systems at farm, slaughter and environment using MLST. An important objective was to determine whether the same *C. coli* population existed in the two production systems, and whether the presence or absence of antimicrobial use selected for a particular population subset. Our aim was to determine (i) the genetic variability or similarity of the ABF and conventional *C. coli* populations, (ii) whether the phenotypic diversity of *C. coli* observed at farm, slaughter, and environment is replicated at the genotypic level, and most importantly (iii) the role played by the environment as a potential reservoir of MDR *C. coli* for pigs at farm and slaughter in the two production systems.

## Materials and Methods

### Origin of *C. coli* Isolates

The *C. coli* isolates were recovered as part of a two year longitudinal study involving pigs reared in two distinct production systems in North Carolina. Pigs reared under the conventional systems received antimicrobials for therapeutic and growth promotion purposes, while in ABF systems there was absence of antimicrobial use. The details regarding antimicrobials administered to conventional pigs, sample collection, *C. coli* prevalence in pigs at farm, slaughter, and their environment, and phenotypic and molecular characterization of isolates were previously reported [11]. Briefly, a total of thirty conventional and eight ABF farms were selected for sampling. Fecal samples from cohorts of 35 pigs and their environment (feed, water, soil, drag swabs, lagoon, and truck swabs) were collected from sows post-farrowing and piglets (7-to-10 days of age), twice at the nursery stage (4 and 7 weeks of age), and twice again at the finishing (16 and 26 weeks of age) stages. Sampling of carcasses from the same cohort of pigs (post-evisceration and post-chill) and mesenteric lymph nodes (MLN) was completed, while the lairage and truck swab samples were collected as part of the slaughter environment.

Overall, the *Campylobacter* prevalence on farm was 69.3% in the pigs (ABF: 72.9%, conventional: 66.6%) and 26.3% in their environment (ABF: 20.5%, conventional: 29.9%). The *Campylobacter* prevalence at slaughter was 23% in carcass (ABF: 27.6%, conventional: 19.8%) and 16.1% at the slaughter plant environment (ABF: 14.8%, conventional: 17.8%). *C. coli* was the predominant *Campylobacter* species identified (99.7%) out of the 2,908 that were isolated. The rest of the isolates (0.3%) corresponded to *C*. *jejuni*.

### Multilocus Sequence Typing

A total of 200 *C. coli* isolates (Table S1) were selected for MLST representing swine and environmental isolates at farm (farrowing, nursery, and finishing stages), and slaughter (carcass and environment) of the ABF (*n* = 100) and conventional (*n* = 100) production systems. Isolate selection was representative of the total number of isolates and antimicrobial susceptibility phenotypes of different swine cohorts at farm, slaughter, and environment. Bacterial DNA was purified with a DNeasy Blood & Tissue kit (Qiagen, Valencia, CA) and quantitated (NanoDrop 2000c; Thermo Scientific Inc., Waltham, MA). DNA purity was assessed based on the A_260_/A_280_ ratio. Seven housekeeping genes (*aspA, glnA, gltA, glyA, pgm, tkt, uncA*) between 402–507 bp were PCR amplified as previously described for *C. coli* MLST typing [17]. PCR products were enzymatically purified (ExoSAP-IT; Affymetrix Inc., Santa Clara, CA) prior to sequencing. Sequencing reactions consisted of 1 µl BigDye (Applied Biosystems, Carlsbad, CA), 1 µl of cleaned PCR product (6–7 ng/µl), 1 µl 3.2 pmol sequencing primer, 2 µl of 5× buffer, and dH_2_O to a final volume of 10 µl. Cycle sequencing of cleaned reactions (CleanSEQ; Beckman Coulter, Brea, CA) was performed in a ABI 3730×l DNA Analyzer (Applied Biosystems), and sequencing parameters consisted of 96°C for 1 min, 25 cycles of 96°C for 10 s, 50°C for 5 s, and 60°C for 4 min. Final hold was at 4°C.

### Genetic Diversity and Phylogenetic Analysis

Sequence data from the ABF and conventional *C. coli* isolates was analyzed and queried against the *Campylobacter* database at http://pubmlst.org/campylobacter for the determination of allelic profiles. New allele sequences and sequence types (STs) were submitted at http://pubmlst.org and incorporated in the *Campylobacter* database. The genetic diversity at individual loci, mean genetic diversity (*H*) and standardized index of association (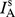) between loci were calculated with LIAN program version 3.5 [22]. Linkage equilibrium, characterized by statistical independence of alleles at all loci and indicative of a freely recombining population, was determined by an 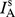
equal to zero. Related STs corresponding to *C. coli* isolates from ABF and conventional production systems were grouped and predicted founders were identified using eBURST v3 algorithm [23]. Groups of STs were defined under a conservative approach by sharing alleles at ≥6 of the 7 loci. The ratio of non-synonymous to synonymous substitutions per site (*d*_N_/*d*_S_) were computed in S.T.A.R.T.2 software [24] using the Nei-Gojobori method. A radial neighbor-joining tree was elaborated with the concatenated allele sequences in MEGA software version 5.0 [25] for the unique STs in the ABF and conventional production systems. BioNumerics version 6.6 (Applied Maths Inc., Austin, TX) was utilized in the construction of a minimum spanning tree with the complete ABF and conventional *C. coli* ST data.

## Results

### Multilocus Sequence Typing (MLST) of *C. coli* Isolates

A total of 51 STs were observed, with unique STs (detected only in specific production systems) in ABF (*n* = 13) and conventional (*n* = 32) *C. coli* isolates. Two novel *C. coli* alleles (*glnA* 424 and *glyA* 464) and 14 novel STs (ABF, *n* = 2; conventional, *n* = 12) were identified and reported for the first time. These new STs were identified at farm, slaughter, and environment of ABF and conventional production systems. Common STs were detected between ABF and conventional *C. coli* isolates at farm, slaughter and/or environment as described in Table 1.

**Table 1:** Common STs between ABF and conventional *C. coli* isolates.

	ST-828 a		ST-854		ST-1068		ST-1096		ST-1185		ST-1186	
Source	ABF (*n* = 3)	Conv (*n* = 4)	ABF(*n* = 31)	Conv (*n* = 17)	ABF(*n* = 12)	Conv (*n* = 6)	ABF(*n* = 1)	Conv (*n* = 3)	ABF (*n* = 5)	Conv (*n* = 3)	ABF(*n* = 17)	Conv(*n* = 1)
Pigs	3 (100)	0	14 (45.2)	3 (17.6)	3 (25)	3 (50)	0	2 (66.7)	3 (60)	3 (100)	6 (35.3)	0
Farm env b	0	2 (50)	14 (45.2)	10 (58.8)	5 (41.7)	3 (50)	0	1 (33.3)	0	0	6 (35.3)	0
Carcass	0	0	3 (9.7)	1 (5.9)	3 (25)	0	0	0	1 (20)	0	4 (23.5)	0
Slaughter env	0	2 (50)	0	3 (17.6)	1 (8.3)	0	1 (100)	0	1	0	1 (5.9)	1 (100)

a No. of isolates (%).

b Environment.

Clonal complex ST-828 was the single lineage identified and was represented by 68% of the ABF and 66% of conventional *C. coli* isolates. ST-854 was the most prevalent ST in both production systems (ABF: 31%, conventional: 17%). STs belonging to ABF *C. coli* isolates were assigned by eBURST to one main group formed by 58 isolates and 11 STs, with ST-828 as the predicted founder of the group. Conventional *C. coli* STs were assigned to two groups. The primary founder for the first group was ST-1096, and the group was constituted by 75 isolates and 22 STs. Alternatively, a presumed founder was not identified for the second group due to the limited number of STs (*n* = 2). Eight ABF and 14 conventional STs were considered as singletons.

### Genetic Diversity of *C. coli* from ABF and Conventional Systems

Genetic diversity at individual loci for the ABF and conventional *C. coli* allelic profiles is shown in Table 2. We detected a mean genetic diversity (*H*) of 0.3963+/−0.0806 and 0.4655+/−0.0714 among *C. coli* isolates from the ABF and conventional production systems, respectively. A greater difference in genetic diversity was observed in the *pgm* (ABF: 0.0396, conventional: 0.3448), *tkt* (ABF: 0.6135, conventional: 0.7042), and *uncA* (ABF: 0.5228, conventional: 0.6121) genes. Allelic diversity was higher in the *glyA* locus of both systems with a greater number of alleles (ABF: 6, conventional: 7). The index of association (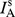) close to zero indicated that the *C. coli* populations were in proximity to linkage equilibrium (ABF: 0.1513, conventional: 0.0991). The non-synonymous to synonymous substitution ratio (*d*_N_/*d*_S_) was determined to be less than one at each locus as shown in Table 2. Testing for selection suggested that nucleotide changes observed did not result in amino acid changes.

**Table 2:** Genetic diversity of *C. coli* from ABF and conventional production systems.

	ABF			Conventional		
Locus	Genetic diversity	Alleles (*n*)	*d*_N_/*d*_S_ a	Genetic diversity	Alleles (*n*)	*d*_N_/*d*_S_
*aspA*	0.1836	3	0.1026	0.2014	3	0.1026
*glnA*	0.5065	3	0.2747	0.5800	5	0.0913
*gltA*	0.3578	2	–	0.2818	4	0.1121
*glyA*	0.5505	6	0.0388	0.5341	7	0.0317
*pgm*	0.0396	2	0	0.3448	4	0
*tkt*	0.6135	5	0.0780	0.7042	6	0.0620
*uncA*	0.5228	3	0	0.6121	4	0
*H* b	0.3963+/−0.0806			0.4655+/−0.0714		
*I* ^s^_A_	0.1513			0.0991		

a Non-synonymous to synonymous ratio.

b Mean genetic diversity.

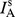
Standardized index of association.

### Distribution of *C. coli* STs and AR Profiles at Farm, Slaughter and Environment

Clonal complex ST-828 consisted of AR and pansusceptible *C. coli* strains from the ABF and conventional production systems. At farm, predominant ST-854 was observed in *C. coli* isolates from five ABF cohorts (A2, A3, A4, A5, and A8) and their environment (Table S1). Within an ABF cohort, ST-854 was found at farm, in swine and environmental isolates, and at slaughter in carcass only. The majority of ABF isolates with ST-854 were associated with MDR profiles (AZI-ERY-TEL-CLI, *n* = 7; and AZI-ERY-TET, *n* = 2), TET resistance (*n* = 16). Conventional isolates assigned to ST-854 were also distributed at farm (*n* = 10) and slaughter (*n* = 3) however, the majority were observed in the respective environments. Conventional *C. coli* isolates with ST-854 predominantly exhibited the MDR phenotype CIP-TET-NAL (*n* = 10).

ST-1186 was mainly distributed in two ABF cohorts (A6 and A7) at farm, from farrowing to finishing, and at slaughter. MDR profiles belonging to ST-1186 were AZI-ERY-TET-TEL (*n* = 14), and AZI-ERY-TET (*n* = 3). Resistance to seven antimicrobials (AZI-CIP-ERY-TET-NAL-TEL-CLI) was observed in ABF *C. coli* isolates (*n* = 3) with ST-5377, which was able to persist from farm to slaughter.

Slaughter carcasses presented unique STs (*n* = 5) absent at the farm and slaughter plant environment. The majority of these STs (*n* = 4) were from *C.coli* strains exhibiting resistance to TET, AZI-ERY-TET-TEL-CLI, AZI-ERY-TET and AZI, ERY, TET, TEL. *C. coli* isolates from ABF lairage presented a greater diversity of STs (*n* = 8) compared to conventional lairage isolates (*n* = 3). Furthermore, half of the ABF lairage STs (*n* = 4) exhibited MDR profiles (ST-1068, AZI-ERY-CLI; ST-1096, AZI-ERY-TEL-CLI; ST-1186, AZI-ERY-TET-TEL; ST-5377, AZI-CIP-ERY-TET-NAL-TEL-CLI). Lairage and truck isolates from the slaughter plant environment of both production systems presented unique STs that were not observed previously at farm (ABF: 4; conventional: 4).

### Phylogenetic Analysis of ABF and Conventional *C. coli* STs

**Figure 1:**
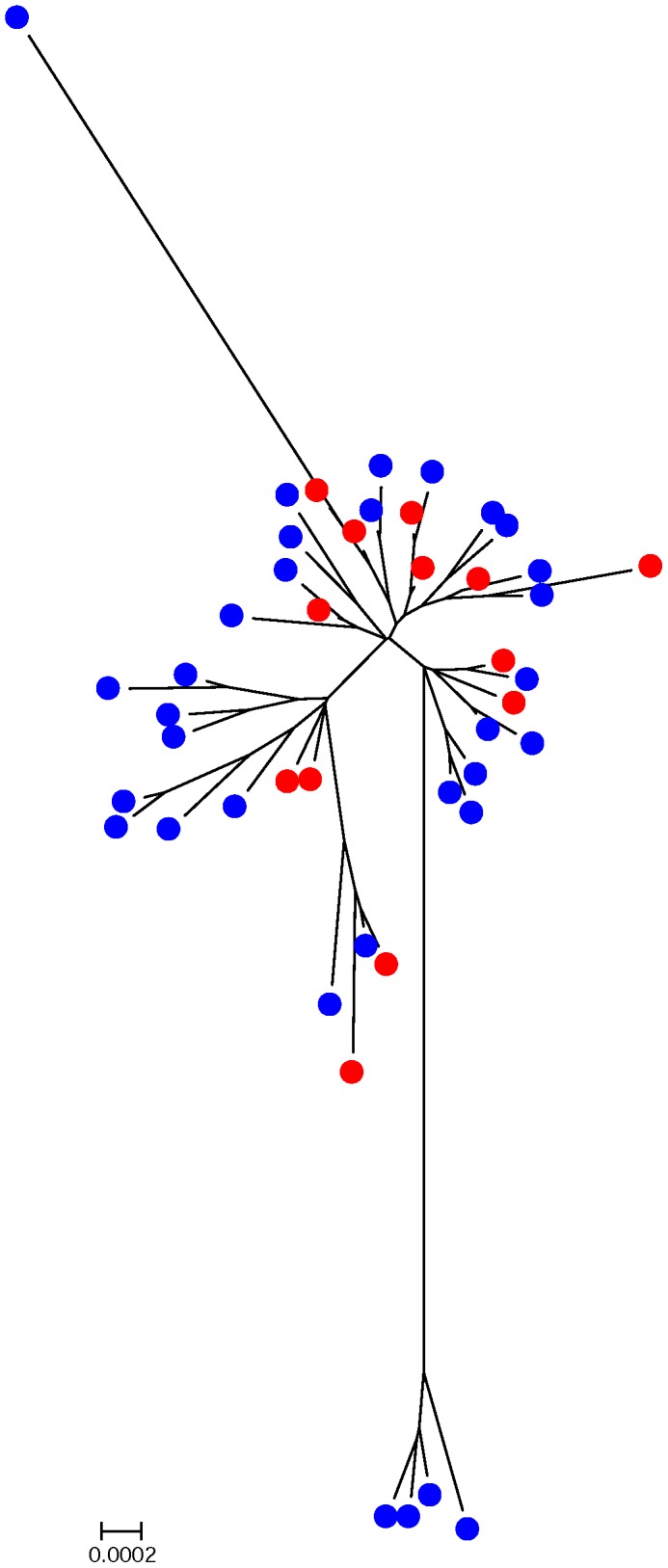
Radial neighbor-joining tree of the unique ABF and conventional *C. coli* STs. ABF and conventional STs are represented by red and blue circles, respectively.

**Figure 2:**
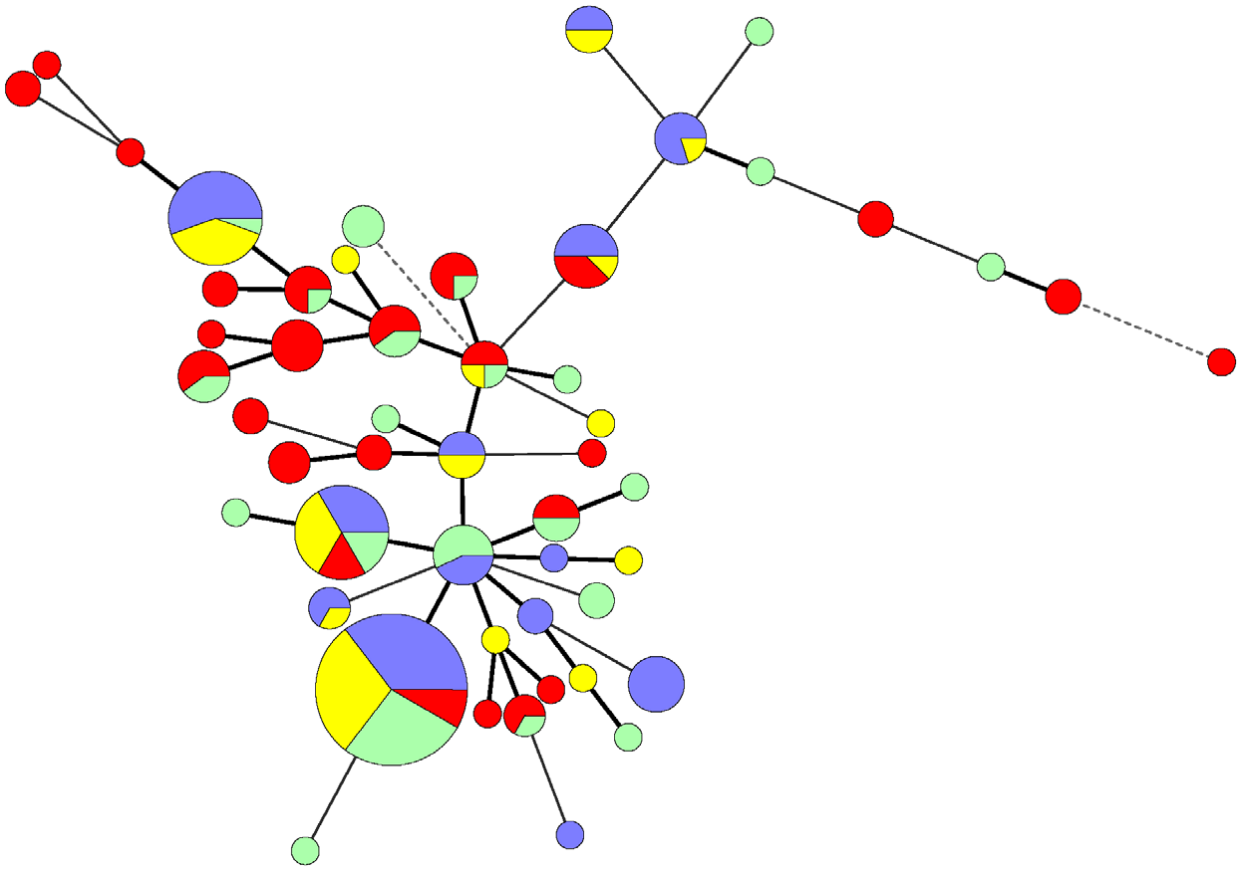
Minimum spanning tree (MST) of *C. coli* isolates from ABF and conventional production systems. Each ST is represented by a node. The size of each node is proportional to the number of strains that comprise that ST. Pie charts with differential colors represent *C. coli* sources: ABF pigs and carcass (purple), ABF environment at farm and slaughter (yellow), conventional pigs and carcass (red), and conventional environment at farm and slaughter (green). Allele differences are represented by thick bold branch lines (single locus variants), thin continuous lines (double locus variants), and dashed lines (three allele differences). Unique STs of the pigs, carcass, and environment at farm and slaughter from ABF and conventional systems were distinguished by single colored nodes.

The evolutionary relationships of the unique ABF (*n* = 13) and conventional (*n* = 32) *C. coli* STs, exclusive to either production system, are represented in a radial neighbor-joining tree (Figure 1). These unique STs were observed in ABF and conventional *C. coli* isolates at farm, slaughter and environment after repeated sampling. The phylogenetic tree displays the close clustering of these ABF and conventional STs with overlap suggesting genetically similar populations. As the tree branch lengths are equivalent to the evolutionary distances, divergence of some of the conventional and ABF *C. coli* STs from the main cluster is observed. This is suggestive of rapidly evolving *C. coli* STs at farm and slaughter. Reference is made to the distant cluster formed by ST-5770, ST-1827, ST-1109, ST-5772, which included only conventional MDR *C. coli* strains. When queried against the MLST database, these *C. coli* STs revealed shared allele’s (*aspA* 33, *glnA* 153, *glnA* 39, and *gltA* 30) with *C. jejuni* isolates from U.S. cattle and humans. Other outlier STs were observed in ABF (ST-5766, ST-1450, and ST-5764) and conventional (ST-5248, ST-1097, and ST-4938) *C. coli* isolates. The minimum spanning tree (Figure 2) created with all the ABF and conventional *C. coli* ST data revealed the close clustering of swine and carcass STs with those of the environment at farm and slaughter, the majority of which exhibited one allele difference. As shown in this figure, several STs (ST-854, ST-1068, ST-1096, ST-1185, ST-1186) shared isolates from farm, slaughter, and environment of ABF and conventional production systems.

## Discussion

There has been extensive debate regarding associations of antimicrobial use in food animals and the development of antimicrobial resistant bacterial strains that can be transferred to humans [7]. In the U.S swine industry antimicrobials are commonly used for therapeutic and non-therapeutic purposes like growth promotion [11], [26]. There has been tremendous pressure on the food animal industry to withdraw the use of sub-therapeutic doses of antimicrobials for growth promotion purposes. A subsequent outcome due to increasing trends in AR bacterial pathogens has been the growth of the organic and ABF food animal industry which prohibits the use of antimicrobials for growth promotion. We recently reported the high prevalence of AR *C. coli* isolated from ABF pigs at farm and slaughter that were never exposed to antimicrobials [11].

Consequently, we aimed to determine the population structure of *C. coli* isolated from ABF reared pigs, and compared it to the *C. coli* population recovered from conventional reared pigs using MLST. The phenotypic diversity of AR *C. coli* from ABF and conventional systems was confirmed at the genotypic level. The linkage equilibrium values of the two *C. coli* swine populations were suggestive of a recombining and weakly clonal population. Based on 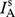
estimates, a lower degree of clonality was observed when compared to results from a previous study (ABF: 0.279, conventional: 0.535) in the same geographic region [3], albeit from different swine farms, no environmental isolates studied, and almost eight years of difference with the present study. The weakly clonal population of *C*. *coli* contrasts with with highly clonal bacterial populations like those of *Escherichia coli* and *Salmonella* [27]. Recombination has proven to be higher in *C. coli* when compared to *C. jejuni* with a more efficient exchange of alleles [28], [29]. The observation of more novel STs compared to alleles also suggests that recombination events are more prone to occur than mutations. The majority of these novel STs belonged to MDR *C. coli* strains at farm, slaughter, and environment. Recombination could explain the genetic diversity observed in AR *C. coli* from ABF and conventional swine systems, which can rapidly occur even in the absence of selective pressure [30].

Clonal complex ST-828 was the single lineage identified in ABF and conventional *C. coli* isolates. This genotypic group seemed to be very well adapted in these two swine productions systems as previously reported [19], with a similar distribution at farm, slaughter, and environment. In the U.S, swine is the main source representing this lineage followed by chickens, cattle and sporadic human cases [19], [31]. Within this lineage is ST-854, a prevalent ST in ABF and conventional *C. coli* isolates, which in the U.S has been exclusively reported in pigs [3], [31]. Niche adaptation of clonal complexes has also been described in *C. jejuni* isolated from humans, poultry, cattle, and sheep [32]. In our study, ST-1096 (member of the ST-828 complex) was identified as the predicted founder for *C. coli* from conventional production systems. This ST is present in pigs, chickens, human stools, and environmental waters (http://pubmlst.org/campylobacter). A high frequency of *C. coli* ST-1068 from ABF swine farms was observed, even though cattle have been reported as the major source of this ST [31]. In the present study, half of the ABF swine farms sampled raised cattle in their fields, representing a potential for the exchange of *C. coli* between cattle and pigs.

Identical STs were observed in *C. coli* isolates from ABF and conventional farm, slaughter, and environment. This was also true between different farms within a production system, suggesting these STs were stable enough to persist and disseminate throughout the different production stages at farm. Pigs from the same cohort also shared the same STs at farm. Novel STs were well adapted to either one of the production systems, while others like ST-854 were detected in both. Sow and piglet STs were diverse enough that genotypic similarities between them were not observed in the majority of the ABF and conventional isolates. This is consistent with previous work where sows and piglets from the same barn did not share the same genotypes by pulse field gel electrophoresis [33]. As previously described [10], associations between ST and AR profile could not be established in our study. *C. coli* isolates with the same ST could exhibit resistance to multiple AR profiles and vice versa. An exception was the ABF MDR pattern resistant to seven antimicrobials AZI-CIP-ERY-TET-NAL-TEL-CLI, which was exclusively observed in isolates with ST- 5377.

We identified diverse STs at the ABF and conventional slaughter plants. In particular, the ABF lairage displayed more STs from AR *C. coli* strains than the conventional lairage. These STs were unique and not observed at the farm level, suggesting the lairage is a potential place for the transmission of AR *C. coli* between pigs from different ABF farms. Unique STs absent at farm or at the slaughter plant environment were also observed in *C. coli* isolates from ABF and conventional swine carcasses at post-evisceration, post-chill, and in MLN samples. It was interesting to note that these diverse *C. coli* STs exhibited varied AR profiles (AZI-ERY-TEL-CLI, AZI-ERY-TET, and AZI-ERY-TET-TEL). Furthermore, some of these STs were present within the same cohort of pigs at different slaughter stages including post-chill, signaling cross-contamination of carcasses with MDR *C. coli* at the slaughter plant level. Genotypes specific to the slaughter plant demonstrate that pigs that do not carry MDR *C. coli* at farm can acquire these AR strains during processing and present them at the final end product. Cross-contamination has also been associated with the slaughtering process of chickens [34]. A study comparing *Campylobacter* genotypes from a chicken flock before and after slaughter revealed a greater diversity at this stage, with 48% of the carcass STs undetected prior to slaughter [35].

As demonstrated by our results, the swine environment plays a key role in the persistence of MDR *C. coli* strains at farm and slaughter despite distinct management practices. This is specifically important when we consider the presence of AR resistant *C. coli* strains in ABF pigs which are raised in the absence of antimicrobial exposure. AR *C. coli* strains were able to persist and disseminate in the absence of antimicrobial selection pressure, likely explained by a common population structure between *C. coli* from conventional and ABF systems. There was a clear overlap of swine and environmental STs, although we cannot ascertain the direction in which *C. coli* was transmitted. Additionally, the diversity of STs present in the environment at farm and slaughter, including unique STs, suggest the environment plays a role in increasing the genetic diversity, as has been previously reported [36].

In conclusion, phylogenetic analysis revealed a genotypically diverse *C. coli* population with the presence of *C. coli* isolates sharing a common ancestry in both production systems.

## Supporting Information

Table S1 **ABF and conventional** ***C. coli*** **isolate source, MLST data, and AR profiles.**
(DOC)
