# Supplementary material for: Phylogenetic Analysis Reveals Common Antimicrobial Resistant Campylobacter coli Population in Antimicrobial-Free (ABF) and Commercial Swine Systems
Source: PLoS One. 2012 Sep 12;7(9):e44662. doi: 10.1371/journal.pone.0044662 (PMC3440321; doi:10.1371/journal.pone.0044662)
Supplement: Table S1 — ABF and conventional C. coli isolate source, MLST data, and AR profiles. (DOC) [file pone.0044662.s001.doc]

Table S1. Isolate source, MLST data, and AR profiles of the ABF and conventional *C*. *coli* isolates

| **Cohort** *^a^* | **Source** *^b^* | **ST** *^c^* | ***aspA*** *^d^* | ***glnA*** | ***gltA*** | ***glyA*** | ***pgm*** | ***tkt*** | ***uncA*** | **CC** *^e^* | **AR Profile** *^f^* |
| --- | --- | --- | --- | --- | --- | --- | --- | --- | --- | --- | --- |
| A1 | N | 828 | 33 | 39 | 30 | 82 | 104 | 43 | 17 | ST-828 | AZI-ERY-TET-TEL-CLI |
| A3 | F | 828 | 33 | 39 | 30 | 82 | 104 | 43 | 17 | ST-828 | TET |
| A1 | FA^*^ | 828 | 33 | 39 | 30 | 82 | 104 | 43 | 17 | ST-828 | AZI-ERY-TET-TEL-CLI |
| C7 | FE | 828 | 33 | 39 | 30 | 82 | 104 | 43 | 17 | ST-828 | CIP-TET-NAL |
| C9 | SE | 828 | 33 | 39 | 30 | 82 | 104 | 43 | 17 | ST-828 | TET |
| C8 | FAE | 828 | 33 | 39 | 30 | 82 | 104 | 43 | 17 | ST-828 | PAN |
| C10 | SE | 828 | 33 | 39 | 30 | 82 | 104 | 43 | 17 | ST-828 | TET |
| A2 | SE | 829 | 33 | 39 | 30 | 82 | 113 | 43 | 17 | ST-828 | TET |
| A7 | NE | 830 | 33 | 39 | 30 | 79 | 104 | 47 | 17 | ST-828 | CIP-TET-NAL |
| A3 | N | 854 | 33 | 38 | 30 | 82 | 104 | 43 | 17 | ST-828 | CIP-TET-NAL |
| A4 | FA | 854 | 33 | 38 | 30 | 82 | 104 | 43 | 17 | ST-828 | AZI-ERY-TEL-CLI |
| A4 | F | 854 | 33 | 38 | 30 | 82 | 104 | 43 | 17 | ST-828 | AZI-ERY-TEL-CLI |
| A4 | NE | 854 | 33 | 38 | 30 | 82 | 104 | 43 | 17 | ST-828 | AZI-ERY-TEL-CLI |
| A4 | FE | 854 | 33 | 38 | 30 | 82 | 104 | 43 | 17 | ST-828 | AZI-ERY-TEL-CLI |
| A4 | NE | 854 | 33 | 38 | 30 | 82 | 104 | 43 | 17 | ST-828 | AZI-ERY-TEL-CLI |
| A2 | FA^*^ | 854 | 33 | 38 | 30 | 82 | 104 | 43 | 17 | ST-828 | TET |
| A2 | F | 854 | 33 | 38 | 30 | 82 | 104 | 43 | 17 | ST-828 | TET |
| A2 | FE | 854 | 33 | 38 | 30 | 82 | 104 | 43 | 17 | ST-828 | TET |
| A2 | NE | 854 | 33 | 38 | 30 | 82 | 104 | 43 | 17 | ST-828 | TET |
| A2 | PE | 854 | 33 | 38 | 30 | 82 | 104 | 43 | 17 | ST-828 | TET |
| A3 | FE | 854 | 33 | 38 | 30 | 82 | 104 | 43 | 17 | ST-828 | AZI-ERY-TET |
| A3 | F | 854 | 33 | 38 | 30 | 82 | 104 | 43 | 17 | ST-828 | AZI-ERY-TET |
| A8 | FA | 854 | 33 | 38 | 30 | 82 | 104 | 43 | 17 | ST-828 | TET |
| A8 | N | 854 | 33 | 38 | 30 | 82 | 104 | 43 | 17 | ST-828 | TET |
| A3 | N | 854 | 33 | 38 | 30 | 82 | 104 | 43 | 17 | ST-828 | TET |
| A8 | NE | 854 | 33 | 38 | 30 | 82 | 104 | 43 | 17 | ST-828 | TET |
| A3 | FE | 854 | 33 | 38 | 30 | 82 | 104 | 43 | 17 | ST-828 | TET |
| A3 | FE | 854 | 33 | 38 | 30 | 82 | 104 | 43 | 17 | ST-828 | TET |
| A5 | N | 854 | 33 | 38 | 30 | 82 | 104 | 43 | 17 | ST-828 | PAN |
| A5 | F | 854 | 33 | 38 | 30 | 82 | 104 | 43 | 17 | ST-828 | PAN |
| A5 | FE | 854 | 33 | 38 | 30 | 82 | 104 | 43 | 17 | ST-828 | PAN |
| A5 | NE | 854 | 33 | 38 | 30 | 82 | 104 | 43 | 17 | ST-828 | PAN |
| A5 | F | 854 | 33 | 38 | 30 | 82 | 104 | 43 | 17 | ST-828 | PAN |
| A4 | MLN | 854 | 33 | 38 | 30 | 82 | 104 | 43 | 17 | ST-828 | AZI-ERY-TEL-CLI |
| A4 | N | 854 | 33 | 38 | 30 | 82 | 104 | 43 | 17 | ST-828 | AZI-ERY-TEL-CLI |
| A2 | MLN | 854 | 33 | 38 | 30 | 82 | 104 | 43 | 17 | ST-828 | TET |
| A8 | F | 854 | 33 | 38 | 30 | 82 | 104 | 43 | 17 | ST-828 | TET |
| A8 | FAE | 854 | 33 | 38 | 30 | 82 | 104 | 43 | 17 | ST-828 | TET |
| A8 | FE | 854 | 33 | 38 | 30 | 82 | 104 | 43 | 17 | ST-828 | TET |
| A3 | FE | 854 | 33 | 38 | 30 | 82 | 104 | 43 | 17 | ST-828 | TET |
| C7 | F | 854 | 33 | 38 | 30 | 82 | 104 | 43 | 17 | ST-828 | CIP-TET-NAL |
| C5 | FE | 854 | 33 | 38 | 30 | 82 | 104 | 43 | 17 | ST-828 | CIP-TET-NAL |
| C5 | FE | 854 | 33 | 38 | 30 | 82 | 104 | 43 | 17 | ST-828 | CIP-TET-NAL |
| C6 | NE | 854 | 33 | 38 | 30 | 82 | 104 | 43 | 17 | ST-828 | CIP-TET-NAL |
| C5 | FE | 854 | 33 | 38 | 30 | 82 | 104 | 43 | 17 | ST-828 | CIP-TET-NAL |
| C5 | FE | 854 | 33 | 38 | 30 | 82 | 104 | 43 | 17 | ST-828 | CIP-TET-NAL |
| C5 | MLN | 854 | 33 | 38 | 30 | 82 | 104 | 43 | 17 | ST-828 | CIP-TET-NAL |
| C10 | SE | 854 | 33 | 38 | 30 | 82 | 104 | 43 | 17 | ST-828 | CIP-TET-NAL |
| C4 | SE | 854 | 33 | 38 | 30 | 82 | 104 | 43 | 17 | ST-828 | CIP-TET-NAL |
| C2 | FE | 854 | 33 | 38 | 30 | 82 | 104 | 43 | 17 | ST-828 | TET |
| C2 | FE | 854 | 33 | 38 | 30 | 82 | 104 | 43 | 17 | ST-828 | TET |
| C2 | FAE | 854 | 33 | 38 | 30 | 82 | 104 | 43 | 17 | ST-828 | TET |
| C1 | FE | 854 | 33 | 38 | 30 | 82 | 104 | 43 | 17 | ST-828 | AZI-ERY-TET |
| C7 | N | 854 | 33 | 38 | 30 | 82 | 104 | 43 | 17 | ST-828 | AZI-CIP-ERY-NAL-TEL-CLI |
| C5 | F | 854 | 33 | 38 | 30 | 82 | 104 | 43 | 17 | ST-828 | CIP-TET-NAL |
| C9 | SE | 854 | 33 | 38 | 30 | 82 | 104 | 43 | 17 | ST-828 | AZI-ERY-TET-TEL |
| C2 | FAE | 854 | 33 | 38 | 30 | 82 | 104 | 43 | 17 | ST-828 | AZI-ERY-TET |
| C7 | FA | 887 | 33 | 38 | 30 | 82 | 104 | 85 | 68 | ST-828 | CIP-TET-NAL |
| C5 | N | 887 | 33 | 38 | 30 | 82 | 104 | 85 | 68 | ST-828 | CIP-TET-NAL |
| C2 | FA^*^ | 887 | 33 | 38 | 30 | 82 | 104 | 85 | 68 | ST-828 | TET |
| C2 | PE | 887 | 33 | 38 | 30 | 82 | 104 | 85 | 68 | ST-828 | TET |
| C3 | FA | 887 | 33 | 38 | 30 | 82 | 104 | 85 | 68 | ST-828 | TET |
| A7 | N | 902 | 33 | 39 | 30 | 79 | 104 | 43 | 17 | ST-828 | AZI-ERY-TET |
| C4 | FA | 1054 | 33 | 39 | 123 | 82 | 104 | 35 | 36 | ST-828 | PAN |
| A1 | FA^*^ | 1058 | 33 | 39 | 30 | 82 | 104 | 35 | 17 | ST-828 | AZI-ERY-TET-TEL-CLI |
| A1 | FE | 1058 | 33 | 39 | 30 | 82 | 104 | 35 | 17 | ST-828 | AZI-ERY-TET-TEL-CLI |
| A1 | F | 1058 | 33 | 39 | 30 | 82 | 104 | 35 | 17 | ST-828 | AZI-ERY-TET-TEL-CLI |
| A1 | NE | 1058 | 33 | 39 | 30 | 82 | 104 | 35 | 17 | ST-828 | AZI-ERY-TET-TEL-CLI |
| C10 | NE | 1059 | 33 | 153 | 30 | 82 | 104 | 35 | 17 | ST-828 | AZI-ERY-TET-TEL |
| A3 | N | 1068 | 33 | 39 | 30 | 78 | 104 | 43 | 17 | ST-828 | AZI-ERY-CLI |
| A5 | F | 1068 | 33 | 39 | 30 | 78 | 104 | 43 | 17 | ST-828 | AZI-ERY-CLI |
| A3 | NE | 1068 | 33 | 39 | 30 | 78 | 104 | 43 | 17 | ST-828 | AZI-ERY-CLI |
| A5 | FE | 1068 | 33 | 39 | 30 | 78 | 104 | 43 | 17 | ST-828 | AZI-ERY-CLI |
| A3 | NE | 1068 | 33 | 39 | 30 | 78 | 104 | 43 | 17 | ST-828 | AZI-ERY-CLI |
| A5 | PE | 1068 | 33 | 39 | 30 | 78 | 104 | 43 | 17 | ST-828 | AZI-ERY-CLI |
| A1 | SE | 1068 | 33 | 39 | 30 | 78 | 104 | 43 | 17 | ST-828 | AZI-ERY-CLI |
| A7 | F | 1068 | 33 | 39 | 30 | 78 | 104 | 43 | 17 | ST-828 | AZI-ERY-TET |
| A1 | FE | 1068 | 33 | 39 | 30 | 78 | 104 | 43 | 17 | ST-828 | PAN |
| A1 | PE | 1068 | 33 | 39 | 30 | 78 | 104 | 43 | 17 | ST-828 | PAN |
| A5 | MLN | 1068 | 33 | 39 | 30 | 78 | 104 | 43 | 17 | ST-828 | PAN |
| A5 | FE | 1068 | 33 | 39 | 30 | 78 | 104 | 43 | 17 | ST-828 | PAN |
| C7 | N | 1068 | 33 | 39 | 30 | 78 | 104 | 43 | 17 | ST-828 | CIP-TET-NAL |
| C7 | NE | 1068 | 33 | 39 | 30 | 78 | 104 | 43 | 17 | ST-828 | CIP-TET-NAL |
| C4 | FA^*^ | 1068 | 33 | 39 | 30 | 78 | 104 | 43 | 17 | ST-828 | PAN |
| C8 | NE | 1068 | 33 | 39 | 30 | 78 | 104 | 43 | 17 | ST-828 | PAN |
| C1 | FA | 1068 | 33 | 39 | 30 | 78 | 104 | 43 | 17 | ST-828 | PAN |
| C10 | FE | 1068 | 33 | 39 | 30 | 78 | 104 | 43 | 17 | ST-828 | TET |
| A3 | SE | 1096 | 33 | 38 | 30 | 82 | 104 | 35 | 17 | ST-828 | AZI-ERY-TEL-CLI |
| C3 | N | 1096 | 33 | 38 | 30 | 82 | 104 | 35 | 17 | ST-828 | TET |
| C10 | NE | 1096 | 33 | 38 | 30 | 82 | 104 | 35 | 17 | ST-828 | AZI-ERY-TET-TEL |
| C4 | FA | 1096 | 33 | 38 | 30 | 82 | 104 | 35 | 17 | ST-828 | AZI-ERY-TET |
| C1 | PE | 1097 | 53 | 38 | 30 | 81 | 104 | 44 | 36 |  | PAN |
| C9 | FA^*^ | 1104 | 33 | 39 | 30 | 82 | 104 | 85 | 68 | ST-828 | AZY-ERY-TET-TEL-CLI |
| C9 | NE | 1104 | 33 | 39 | 30 | 82 | 104 | 85 | 68 | ST-828 | AZY-ERY-TET-TEL-CLI |
| C5 | PE | 1104 | 33 | 39 | 30 | 82 | 104 | 85 | 68 | ST-828 | CIP-TET-NAL |
| C9 | FE | 1104 | 33 | 39 | 30 | 82 | 104 | 85 | 68 | ST-828 | AZI-ERY-TET-TEL |
| C9 | F | 1104 | 33 | 39 | 30 | 82 | 104 | 85 | 68 | ST-828 | AZY-ERY-TET-TEL-CLI |
| A5 | SE | 1107 | 33 | 38 | 30 | 82 | 104 | 44 | 36 | ST-828 | PAN |
| C10 | FA^*^ | 1108 | 33 | 39 | 30 | 78 | 113 | 43 | 17 | ST-828 | AZI-ERY-TET-TEL |
| C5 | N | 1109 | 33 | 38 | 30 | 173 | 104 | 35 | 68 |  | AZY-ERY-TET-TEL-CLI |
| C7 | FE | 1109 | 33 | 38 | 30 | 173 | 104 | 35 | 68 |  | CIP-TET-NAL |
| C7 | N | 1109 | 33 | 38 | 30 | 173 | 104 | 35 | 68 |  | AZI-CIP-ERY-TET-NAL-TEL |
| C5 | FA | 1109 | 33 | 38 | 30 | 173 | 104 | 35 | 68 |  | AZY-ERY-TET-TEL-CLI |
| A4 | FE | 1130 | 33 | 38 | 30 | 82 | 104 | 43 | 68 | ST-828 | AZI-ERY-TET-TEL |
| C5 | FA^*^ | 1143 | 32 | 38 | 44 | 82 | 104 | 43 | 36 |  | AZY-ERY-TET-TEL-CLI |
| C3 | FA^*^ | 1143 | 32 | 38 | 44 | 82 | 104 | 43 | 36 |  | TET |
| A1 | PE | 1145 | 33 | 39 | 30 | 82 | 104 | 44 | 17 | ST-828 | AZI-ERY-TET-TEL-CLI |
| A1 | FA^*^ | 1145 | 33 | 39 | 30 | 82 | 104 | 44 | 17 | ST-828 | PAN |
| C5 | FE | 1177 | 33 | 38 | 30 | 82 | 104 | 85 | 17 | ST-828 | CIP-TET-NAL |
| A3 | PE | 1185 | 33 | 38 | 44 | 82 | 104 | 35 | 36 |  | TET |
| A3 | SE | 1185 | 33 | 38 | 44 | 82 | 104 | 35 | 36 |  | TET |
| A3 | FA | 1185 | 33 | 38 | 44 | 82 | 104 | 35 | 36 |  | TET |
| A2 | N | 1185 | 33 | 38 | 44 | 82 | 104 | 35 | 36 |  | TET |
| A3 | FA^*^ | 1185 | 33 | 38 | 44 | 82 | 104 | 35 | 36 |  | TET |
| C10 | FA | 1185 | 33 | 38 | 44 | 82 | 104 | 35 | 36 |  | AZI-ERY-TET-TEL |
| C10 | F | 1185 | 33 | 38 | 44 | 82 | 104 | 35 | 36 |  | AZI-ERY-TET-TEL |
| C10 | N | 1185 | 33 | 38 | 44 | 82 | 104 | 35 | 36 |  | AZI-ERY-TET-TEL |
| A6 | FA^*^ | 1186 | 33 | 38 | 30 | 173 | 104 | 35 | 36 |  | AZI-ERY-TET-TEL |
| A7 | FA^*^ | 1186 | 33 | 38 | 30 | 173 | 104 | 35 | 36 |  | AZI-ERY-TET-TEL |
| A6 | MLN | 1186 | 33 | 38 | 30 | 173 | 104 | 35 | 36 |  | AZI-ERY-TET-TEL |
| A6 | FE | 1186 | 33 | 38 | 30 | 173 | 104 | 35 | 36 |  | AZI-ERY-TET-TEL |
| A6 | NE | 1186 | 33 | 38 | 30 | 173 | 104 | 35 | 36 |  | AZI-ERY-TET-TEL |
| A7 | FE | 1186 | 33 | 38 | 30 | 173 | 104 | 35 | 36 |  | AZI-ERY-TET-TEL |
| A6 | NE | 1186 | 33 | 38 | 30 | 173 | 104 | 35 | 36 |  | AZI-ERY-TET-TEL |
| A6 | N | 1186 | 33 | 38 | 30 | 173 | 104 | 35 | 36 |  | AZI-ERY-TET-TEL |
| A7 | N | 1186 | 33 | 38 | 30 | 173 | 104 | 35 | 36 |  | AZI-ERY-TET-TEL |
| A6 | F | 1186 | 33 | 38 | 30 | 173 | 104 | 35 | 36 |  | AZI-ERY-TET-TEL |
| A7 | F | 1186 | 33 | 38 | 30 | 173 | 104 | 35 | 36 |  | AZI-ERY-TET-TEL |
| A6 | PE | 1186 | 33 | 38 | 30 | 173 | 104 | 35 | 36 |  | AZI-ERY-TET-TEL |
| A7 | PE | 1186 | 33 | 38 | 30 | 173 | 104 | 35 | 36 |  | AZI-ERY-TET-TEL |
| A7 | FE | 1186 | 33 | 38 | 30 | 173 | 104 | 35 | 36 |  | AZI-ERY-TET |
| A7 | NE | 1186 | 33 | 38 | 30 | 173 | 104 | 35 | 36 |  | AZI-ERY-TET |
| A6 | SE | 1186 | 33 | 38 | 30 | 173 | 104 | 35 | 36 |  | AZI-ERY-TET-TEL |
| A7 | PE | 1186 | 33 | 38 | 30 | 173 | 104 | 35 | 36 |  | AZI-ERY-TET |
| C7 | SE | 1186 | 33 | 38 | 30 | 173 | 104 | 35 | 36 |  | PAN |
| C6 | F | 1197 | 33 | 39 | 30 | 82 | 118 | 35 | 68 | ST-828 | AZI-CIP-ERY-TET-NAL-TEL |
| C2 | PC | 1197 | 33 | 39 | 30 | 82 | 118 | 35 | 68 | ST-828 | TET |
| C4 | F | 1197 | 33 | 39 | 30 | 82 | 118 | 35 | 68 | ST-828 | AZI-ERY-TET |
| C9 | FE | 1429 | 33 | 39 | 44 | 82 | 189 | 35 | 36 |  | AZY-ERY-TET-TEL-CLI |
| A1 | SE | 1438 | 33 | 153 | 30 | 82 | 104 | 44 | 17 | ST-828 | PAN |
| C1 | FE | 1444 | 33 | 39 | 30 | 82 | 104 | 171 | 17 | ST-828 | AZI-ERY-TET |
| C4 | MLN | 1444 | 33 | 39 | 30 | 82 | 104 | 171 | 17 | ST-828 | PAN |
| C4 | MLN | 1444 | 33 | 39 | 30 | 82 | 104 | 171 | 17 | ST-828 | AZI-ERY-TET |
| C1 | NE | 1444 | 33 | 39 | 30 | 82 | 104 | 171 | 17 | ST-828 | PAN |
| A1 | PC | 1450 | 53 | 39 | 44 | 82 | 104 | 35 | 36 |  | PAN |
| A1 | FE | 1450 | 53 | 39 | 44 | 82 | 104 | 35 | 36 |  | PAN |
| A1 | PC | 1450 | 53 | 39 | 44 | 82 | 104 | 35 | 36 |  | PAN |
| A1 | F | 1450 | 53 | 39 | 44 | 82 | 104 | 35 | 36 |  | PAN |
| A1 | MLN | 1450 | 53 | 39 | 44 | 82 | 104 | 35 | 36 |  | PAN |
| C2 | MLN | 1750 | 33 | 39 | 30 | 115 | 113 | 43 | 17 | ST-828 | TET |
| C7 | MLN | 1750 | 33 | 39 | 30 | 115 | 113 | 43 | 17 | ST-828 | CIP-TET-NAL |
| C8 | NE | 1750 | 33 | 39 | 30 | 115 | 113 | 43 | 17 | ST-828 | PAN |
| C6 | F | 1827 | 33 | 39 | 30 | 173 | 113 | 35 | 68 |  | AZI-ERY-TEL-CLI |
| C6 | MLN | 1827 | 33 | 39 | 30 | 173 | 113 | 35 | 68 |  | AZI-ERY-TEL-CLI |
| C2 | PC | 1834 | 33 | 39 | 30 | 82 | 104 | 35 | 68 | ST-828 | AZI-ERY-TET |
| C2 | PE | 1834 | 33 | 39 | 30 | 82 | 104 | 35 | 68 | ST-828 | AZI-ERY-TET |
| C2 | F | 1836 | 32 | 160 | 44 | 82 | 118 | 44 | 36 |  | TET |
| C2 | PC | 1836 | 32 | 160 | 44 | 82 | 118 | 44 | 36 |  | TET |
| C5 | FE | 2508 | 33 | 38 | 30 | 82 | 104 | 35 | 68 | ST-828 | CIP-TET-NAL |
| C2 | N | 2508 | 33 | 38 | 30 | 82 | 104 | 35 | 68 | ST-828 | TET |
| C2 | FAE | 2508 | 33 | 38 | 30 | 82 | 104 | 35 | 68 | ST-828 | TET |
| C1 | N | 2508 | 33 | 38 | 30 | 82 | 104 | 35 | 68 | ST-828 | AZI-ERY-TET |
| C1 | N | 2508 | 33 | 38 | 30 | 82 | 104 | 35 | 68 | ST-828 | AZI-CIP-ERY-TET-NAL-CLI |
| A6 | N | 2877 | 33 | 39 | 44 | 82 | 104 | 44 | 36 | ST-828 | AZI-ERY-GEN-TET-NAL-TEL-CLI |
| A7 | PE | 2877 | 33 | 39 | 44 | 82 | 104 | 44 | 36 | ST-828 | AZI-ERY-TET-TEL-CLI |
| A7 | N | 2877 | 33 | 39 | 44 | 82 | 104 | 44 | 36 | ST-828 | AZI-ERY-TET-TEL-CLI |
| A7 | FA | 2877 | 33 | 39 | 44 | 82 | 104 | 44 | 36 | ST-828 | AZI-ERY-TET-TEL-CLI |
| A7 | FA | 2877 | 33 | 39 | 44 | 82 | 104 | 44 | 36 | ST-828 | AZI-ERY-TET-TEL |
| A7 | F | 2877 | 33 | 39 | 44 | 82 | 104 | 44 | 36 | ST-828 | AZI-ERY-TET-TEL-CLI |
| C3 | PE | 4609 | 33 | 38 | 44 | 81 | 118 | 35 | 36 |  | TET |
| C3 | F | 4609 | 33 | 38 | 44 | 81 | 118 | 35 | 36 |  | TET |
| C9 | NE | 4938 | 53 | 39 | 44 | 82 | 104 | 43 | 36 | ST-828 | AZY-ERY-TET-TEL-CLI |
| C3 | NE | 5248 | 33 | 38 | 134 | 161 | 104 | 47 | 17 |  | TET |
| C4 | FAE | 5248 | 33 | 38 | 134 | 161 | 104 | 47 | 17 |  | PAN |
| C4 | FE | 5248 | 33 | 38 | 134 | 161 | 104 | 47 | 17 |  | PAN |
| A2 | N | 5377 | 33 | 39 | 44 | 82 | 104 | 43 | 68 | ST-828 | AZI-CIP-ERY-TET-NAL-TET-CLI |
| A2 | F | 5377 | 33 | 39 | 44 | 82 | 104 | 43 | 68 | ST-828 | AZI-CIP-ERY-TET-NAL-TET-CLI |
| A3 | SE | 5377 | 33 | 39 | 44 | 82 | 104 | 43 | 68 | ST-828 | AZI-CIP-ERY-TET-NAL-TET-CLI |
| C2 | FE | 5745 | 33 | 38 | 30 | 464 | 104 | 35 | 17 | ST-828 | TET |
| C2 | FA | 5745 | 33 | 38 | 30 | **464** | 104 | 35 | 17 | ST-828 | TET |
| A1 | N | 5766 | 53 | 39 | 44 | 174 | 104 | 44 | 36 |  | PAN |
| C4 | F | **5745** | 33 | 38 | 30 | **464** | 104 | 35 | 17 | ST-828 | PAN |
| C7 | PE | **5745** | 33 | 38 | 30 | **464** | 104 | 35 | 17 | ST-828 | CIP-TET-NAL |
| C4 | N | **5746** | 33 | 38 | 30 | 81 | 104 | 35 | 36 |  | PAN |
| A4 | PE | **5764** | 32 | 39 | 30 | 115 | 113 | 85 | 17 |  | AZI-ERY-TEL-CLI |
| A1 | FA | **5766** | 53 | 39 | 44 | 174 | 104 | 44 | 36 |  | PAN |
| A1 | FE | **5766** | 53 | 39 | 44 | 174 | 104 | 44 | 36 |  | PAN |
| A1 | NE | **5766** | 53 | 39 | 44 | 174 | 104 | 44 | 36 |  | PAN |
| C3 | SE | **5767** | 33 | 39 | 30 | 82 | 118 | 171 | 17 |  | TET |
| C3 | MLN | **5768** | 33 | **424** | 30 | 82 | 113 | 43 | 17 |  | TET |
| C3 | NE | **5769** | 32 | 153 | 30 | 82 | 104 | 43 | 17 |  | TET |
| C3 | NE | **5769** | 32 | 153 | 30 | 82 | 104 | 43 | 17 |  | TET |
| C8 | MLN | **5770** | 33 | 153 | 30 | 173 | 104 | 35 | 68 |  | AZI-ERY-TET-TEL |
| C8 | PE | **5770** | 33 | 153 | 30 | 173 | 104 | 35 | 68 |  | AZI-ERY-TET-TEL |
| C5 | SE | **5771** | 32 | 160 | 44 | 82 | 118 | 43 | 36 |  | PAN |
| C7 | FA | **5772** | 33 | 38 | 30 | 173 | 104 | 85 | 68 |  | CIP-TET-NAL |
| C9 | SE | **5773** | 33 | 153 | 30 | 82 | 113 | 44 | 17 |  | TET |
| C3 | FAE | **5774** | 33 | 39 | 30 | 78 | 118 | 43 | 17 |  | TET |
| C8 | FE | **5775** | 32 | 38 | 30 | 82 | 104 | 43 | 42 |  | AZI-ERY-TET-TEL |
| C6 | FA^*^ | **5776** | 32 | 39 | 30 | 81 | 118 | 44 | 36 |  | AZI-ERY-TEL-CLI |

*^a^* ABF cohorts, A1-A8; conventional cohorts, C1-C10.

*^b^* FA, piglets at farrowing; FA^*^, sows at farrowing; FAE, farrowing environment; N, nursery; NE, nursery environment; F, finishing; FE, finishing environment; PE, post-evisceration; MLN, mesenteric lymph nodes; PC, post-chill; SE, slaughter plant environment.

*^c^* Sequence type. *^d^* Allele number.  *^e^* Clonal complex. Novel allele number or STs are represented in bold.

*^f^* Antimicrobial resistance profile: TET, tetracycline; AZI, azithromycin; ERY, erythromycin; TEL, telithromycin; CLI, clindamycin; CIP, ciprofloxacin; NAL, nalidixic acid; GEN, gentamicin; PAN, pansusceptible.
